# Impact of Drug–Drug Interactions on Clinical Outcomes in Metastatic Melanoma Patients Treated With Combined BRAF/MEK Inhibitors: A Real‐World Study

**DOI:** 10.1111/pcmr.70026

**Published:** 2025-06-01

**Authors:** Silvia Mezi, Andrea Botticelli, Giulia Pomati, Simone Scagnoli, Giulia Fiscon, Federica de Galitiis, Francesca Romana di Pietro, Sofia Verkhovskaia, Sasan Amirhassankhani, Giovanna Gentile, Maurizio Simmaco, Bjoern Gohlke, Robert Preissner, Daniele Santini, Paolo Marchetti

**Affiliations:** ^1^ Department of Radiological Science, Oncology and Anatomical Pathology, Faculty of Medicine and Dentistry “Sapienza University of Rome” and Unit Medical Oncology Policlinico Umberto I University Hospital Rome Italy; ^2^ Department of Molecular Medicine Sapienza University of Rome Rome Italy; ^3^ Department of Computer, Control, and Management Engineering “Antonio Ruberti” “Sapienza” University of Rome Rome Italy; ^4^ Istituto Dermopatico Dell'immacolata, IDI‐IRCCS Rome Italy; ^5^ Department of Urology S. Orsola‐Malpighi Hospital University of Bologna Bologna Italy; ^6^ Department of Neuroscience, Mental Health, and Sensory Organs (NESMOS), Faculty of Medicine and Psychology Sapienza University Rome Italy; ^7^ Unit of Laboratory and Advanced Molecular Diagnostics Sant'Andrea University Hospital Rome Italy; ^8^ Structural Bioinformatics Group, Institute for Physiology Charité‐University Medicine Berlin Berlin Germany; ^9^ Department of Medico‐Surgical Sciences and Biotechnology, Polo Pontino Sapienza University of Rome Rome Italy

**Keywords:** BRAF inhibitors, BRAF mutation, drug–drug interaction, MEK inhibitors, metastatic melanoma, outcome, prognostic factors

## Abstract

The unique pharmacokinetics of BRAF and MEK inhibitors make patients vulnerable to drug–drug interactions (DDIs), which may compromise treatment efficacy in metastatic melanoma. This study evaluates the impact of DDIs on clinical outcomes in patients with metastatic melanoma treated with BRAF/MEK inhibitors. This multicenter, observational, retrospective study assessed DDIs using the Drug‐PIN software. Associations between the Drug‐PIN continuous score, Drug‐PIN light, and treatment outscomes were analyzed along with the specific drugs involved in the DDIs. A total of 177 patients with BRAF‐mutant metastatic melanoma undergoing BRAF/MEK inhibitor therapy were included. Of these, 94 patients (55.9%) were exposed to complex drug regimens related to comorbidities, supportive care, and symptom management. A significant change in Drug‐PIN scores was observed before and after therapy initiation. Patients with low‐grade DDIs demonstrated significantly longer median overall survival (OS) and progression‐free survival (PFS) compared to those with high‐grade DDIs (log‐rank *p* = 0.0045 and *p* = 0.012, respectively); this observation was further validated by multiple regression analysis. By combining clinical and DDI data, we identified four patient subgroups with distinct prognoses, showing statistically significant differences in OS and PFS (log‐rank *p* < 0.0001). The subgroup with the highest clinical risk and high DDI had markedly poorer outcomes (HR 2.87, 95% CI [1.7–4.8], *p* < 0.001). The drugs involved in high‐level pharmacological interactions were analyzed. DDIs significantly contribute to poorer OS and PFS outcomes, independent of other clinical risk factors. Optimizing pharmacological regimens to minimize DDIs should be prioritized to enhance treatment efficacy in oncology. Prospective clinical trials are warranted to further validate the advantages of individualized, preemptive therapy optimization.

## Graphical abstract

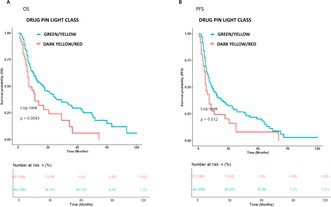

**Summary**

- The pharmacokinetics of BRAF and MEK inhibitors increase patient susceptibility to drug–drug interactions (DDIs). This multicenter retrospective study utilized the Drug‐PIN software to assess DDIs in 177 patients with metastatic melanoma.
- DDIs in BRAF/MEK inhibitor therapy can undermine treatment effectiveness, with higher‐grade DDIs significantly associated with reduced overall survival (OS) and progression‐free survival (PFS). Patients with low‐grade DDIs experienced markedly better survival outcomes compared to those with high‐grade DDIs. Four distinct risk subgroups, delineated through combined clinical and DDI data, exhibited statistically significant differences in prognosis.
- DDIs independently influence clinical outcomes beyond other risk factors, emphasizing the necessity of optimizing therapeutic regimens to mitigate DDIs and improve treatment efficacy.

## 1 Introduction

Approximately half of the patients suffering from cutaneous melanoma (M) harbor an activating mutation at codon 600, predominant in exon 15 (V600E) in the serine–threonine kinase BRAF (BRAF^v600^) (Goydos et al. 2005; Panka et al. 2006). BRAF^v600^ mutations constitutively activate BRAF and upregulate the downstream signal transduction in the RAS/MAPK pathway, which, in turn, regulates cell growth, differentiation, migration, and apoptosis, affecting fundamental processes in melanoma cells, including carcinogenesis, proliferation, progression, and immune escape (Maurer et al. 2011; Ascierto et al. 2012). The potent and selective inhibitors of BRAF‐mutant kinase vemurafenib (V), dabrafenib (D), and encorafenib (E) have demonstrated an advantage in survival outcomes in untreated BRAF^v600^ mutant metastatic melanoma (MM) compared with standard chemotherapy (Chapman et al. 2017, 2011). Nevertheless, the landmark clinical trials demonstrate a rapid development of resistance to BRAF^v600^ inhibitors (BRAF‐i), as well as the development of secondary early squamous cell carcinoma induced by a paradoxical overactivation of the MEK‐mediated signaling cascade occurring both in melanoma cancer cells and in normal cells, in which this pathway is essential for tumor development (Flaherty et al. 2012; Carnahan et al. 2010; Sosman et al. 2012; Blank et al. 2017; Ascierto et al. 2013; Long et al. 2015). The addition of MEK inhibitors (MEK‐i), a signaling molecule downstream of BRAF, has been shown to enhance the activity of BRAF‐i significantly, delaying the development of biological resistance (Robert et al. 2015; Larkin et al. 2014; Ascierto et al. 2016; Dummer et al. 2017). The small molecules trametinib (T), cobimetinib (C), and binimetinib (B) have been extensively investigated in association with BRAF‐i in large first‐line phase III trials enrolling untreated MM patients with BRAF^v600^ mutation (Robert et al. 2015; Dummer et al. 2022; Ascierto et al. 2016), improving progression‐free survival (PFS) and overall survival (OS) as well as the response rate (RR) versus single‐agent therapy (Dummer et al. 2018; Malki and Pearson 2020).

Metabolism of BRAF‐i/MEK‐i drugs involves the cytochromes of the P450 (CYPs) enzyme superfamily, which play a central role in the occurrence of drug interaction, as well as P‐glycoprotein, ATP‐binding cassette transporters, and the detoxifying and DNA‐repair enzymes. Thus, the peculiar pharmacokinetics of BRAF‐i and MEK‐i () expose patients to a potential and significant risk of drug–drug interactions (DDI) when concomitant medications are taken. Numerous categories of concomitant drugs, which are either substrates, inhibitors, or inducers of the same cytochromes, are involved, and drug–drug interactions are expected (Centanni et al. 2019). Moreover, food intake, dietary supplements, complementary alternative therapies, and excipients can interfere with the pharmacodynamics and pharmacokinetics of BRAF‐i/MEK‐i combinations (Beijnen and Schellens 2004). Environmental factors affect the absorption, distribution, metabolism, and excretion of drugs as well; these could also be affected by interpatient variability, given the potential impact of age, gender, pharmacogenetics, and comorbidity conditions on drug handling (Fatunde and Brown 2020; Scripture and Figg 2006; Tannenbaum and Sheehan 2014; Lee et al. 2019; Budha et al. 2016; Dong et al. 2021). In this complex scenario, the metabolic interactions influencing bioavailability and the plasma concentrations of drugs could alter the expected drug disposition in either a beneficial or a harmful manner, reducing treatment efficacy or promoting the development of adverse drug reactions. At the same time, DDIs can interfere with the bioavailability of home medications, exposing patients to subeffective treatments of their concomitant pathologies (Palleria et al. 2013). Although the expected risk of DDIs is significant and quite common, to date, DDIs in cancer treatment have been investigated only in a few retrospective studies, and among them, no patients with MM treated with target therapy were included (van Leeuwen et al. 2013, 2011; Riechelmann et al. 2007).

This multicenter retrospective study evaluates the impact of DDIs in a real‐world population undergoing treatment with BRAFi and MEKi. Our initial safety analysis revealed a significant correlation between the presence of DDIs and an increased incidence of adverse toxicities among patients. Notably, cardiovascular toxicity has emerged as a critical safety concern, and our findings indicate that DDIs represent an additional risk factor for the development of cardiological adverse reactions during BRAFi and MEKi therapy (Mezi et al. 2023). In the present study, we analyze patient outcomes within the same patient population to evaluate the potential adverse effects of DDIs on the efficacy of BRAFi and MEKi therapy. Additionally, we analyze the drugs involved in high‐grade interactions among patients receiving treatment with BRAFi and MEKi therapy.

The DDIs were analyzed using Drug‐PIN (Personalized Interactions Network) a medical software designed to identify drug interactions based on established pharmacokinetic and pharmacodynamic data for each drug. This software integrates demographic (age, gender, ethnicity), clinical, and biochemical data of patients to provide a comprehensive analysis (Sharma et al. 2019; Palleria et al. 2013). Thus, the Drug‐PIN tool includes the full spectrum of variables influencing drug response: the pharmacogenomic profile, DDI, biochemical profile, such as renal and hepatic function, and environmental factors (cigarette smoking habits, alcohol consumption, and caffeine intake).

The goal of this study was to retrospectively assess DDIs to define the risk of drug interactions in clinical practice and their impact on the outcomes of patients with BRAF^v600^ mutant MM treated with BRAF‐i/MEK‐i inhibitors.

An additional objective of this study is to identify drugs with a high potential for interaction with BRAF‐i and MEK inhibitors MEK‐I based on the Drug‐PIN score report resulting from their combination. Furthermore, the study aims to elucidate the underlying pharmacological mechanisms of these interactions. It is equally important to identify which drugs are considered safe for use alongside BRAF‐i and MEK‐i, owing to their low potential for pharmacological interaction. Finally, establishing pharmacological alternatives to drugs with a higher potential for interaction is clinically valuable, as it may facilitate substitution, mitigate interaction‐related toxicity, reduce the impact of therapy on outcome and quality of life, and decrease the necessity for treatment interruptions.

Reducing the effects of drug interactions may critically maximize outcome and minimize toxicity, implementing a personalized strategic approach that is the basis of precision medicine.

## 2 Methods and Materials

### 2.1 Patients and Treatments

As previously reported (Mezi et al. 2023) this observational, multicenter, retrospective study was conducted, including patients with BRAF^v600^ MM who received at least 1 month of BRAF‐i/MEK‐i combination therapy from January 2018 to October 2021. Clinical data were collected from “Policlinico Umberto I – Sapienza” University of Rome and “Istituto Dermopatico dell'Immacolata” (IDI) in Rome. All patients have received D/T, E/B, or V/C at standard doses until either disease progression or unacceptable toxicities were recorded in the first or subsequent lines. Dose reduction levels were collected and reported. Full availability of data concerning patient clinical characteristics, comorbidities, concomitant medications, and oncological outcomes was required as additional inclusion criteria. The following clinical data were retrospectively collected: Eastern Cooperative Oncology Group (ECOG) Performance Status (PS), age, gender, comorbidity, concomitant medication, previous treatments, current line of treatment, metastatic sites, and baseline LDH levels. Based on at least one of the following established prognostic features, a prognostic risk category of high (Risk‐H) or low (Risk‐L) was defined for each patient: lactate dehydrogenase (LDH) > 2UNL, number of metastases ≥ 3, and presence of brain metastases (Sperduto et al. 2017; Weide et al. 2013; Nieder and Mehta 2009).

Clinical and instrumental evaluations were performed every three months on each patient. Written informed consent was obtained from living patients to process their clinical data anonymously.

The study was conducted in accordance with the Declaration of Helsinki, and the protocol was approved by the Ethics Committee of the Coordinating Center (Policlinico Umberto I Prot. 0435/2021 Rif. 6332).

### 2.2 Treatment Outcomes

All patients were assessed for treatment outcomes. PFS was defined as the time from the beginning of the target therapy treatment to disease progression or death. OS was defined as the time from the start of treatment to death. For PFS and OS, patients without events or lost to follow‐up were considered censored at the time of the last follow‐up. Through the Response Evaluation Criteria in Solid Tumors (RECIST), the best tumor response was assessed and classified as complete response (CR), partial response (PR), stable disease (SD), and progressive disease (PD). Based on the response to target therapy, patients were classified as non‐responders if PD occurred at the first clinical‐instrumental evaluation after starting combination therapy or responders if either PR, CR, or at least SD was obtained.

### 2.3 Assessment of Drug Interactions

DDIs were assessed using Drug‐PIN software (https://www.drug‐PIN.com/). A Drug‐PIN score of DDIs based on multiple patient drug interactions was performed for each patient.

The extent of the interaction is quantified by a numerical value, referred to as the Drug‐PIN score, and is represented using a color‐coding system. This system ranges from green (indicating a low‐risk drug cocktail, with a score range of 0–20) to yellow (score range of 21–30), and from dark yellow to red (representing a high‐risk drug cocktail, with a score > 31) [38]. For each patient, the degree of interaction was subsequently classified as either low (DDI‐L), which corresponds to a Drug‐PIN score ranging from 0 to 30 and is indicated by a color light from green to yellow, or high (DDI‐H), which corresponds to a Drug‐PIN score greater than 31 and is indicated by a color light from dark yellow to red. During the score calculation, Drug‐PIN classifies each drug according to its pharmacological class and provides alternative drugs within the same class. It then estimates the Drug‐PIN score for each alternative. Once the Drug‐PIN score is calculated, the software generates a report detailing the contribution of each drug to the final score, considering the patient's age and metabolism. This report outlines the hepatic enzymes involved in metabolizing the drugs for each patient, indicating whether they are substrates, inducers, and/or inhibitors of these enzymes. It also highlights potential interactions for each therapeutic combination, such as competition for the same enzyme, coexisting substrates and inhibitors (which may increase systemic exposure), or substrates and inducers (which may reduce systemic exposure).

To evaluate the drugs associated with DDIs between targeted therapy with BRAFi and MEKi and the home medications, patients with DDI‐H were assessed. An analysis of the co‐administered drugs and their impact on pharmacological interactions was conducted.

### 2.4 Statistical Analysis

The correlations between low or high levels of DDI and the clinical risk category, therapy response, OS and PFS, and cumulative survival rates were computed using the Kaplan–Meier method (Rich et al. 2010). The log‐rank test was used to compare the survival outcomes among the different patient groups categorized by the dichotomization into DDI‐L and DDI‐H interactions or by the risk category (low‐risk vs. high‐risk) or therapeutic response. A log‐rank *p*‐value equal to or less than 0.05 was considered statistically significant: the lower the *p*‐value, the better the separation between the two prognostic groups. A multiple logistic regression model was then applied to evaluate the prediction of therapeutic response based on the combination of various predictor variables (e.g., age, gender, metastasis, the grade of drug–drug interactions (DDI‐L vs. DDI‐H), therapy lines, and comorbidity). A Cox multiple logistic regression model was then computed to test if those different predictor variables or covariates potentially affect patient prognosis regarding OS or PFS and to offer estimates of the strength of effect for each constituent factor. The Wald test for the multiple regression model was used to test whether the predictor variables significantly differ from zero. R software was used to elaborate on the data collected.

## 3 Results

### 3.1 Patients

One hundred seventy‐seven patients with BRAF^v600^MM were enrolled in the study. Their clinical‐pathological features are reported in Table 1. Almost half of the patients (90, 50.8%) had lung metastases, 83 patients (46.9%) had liver metastases, and 99 patients (55.9%) had node locations. Furthermore, 44 patients had brain metastases (24.8%). Seventy‐two patients (40.7%) had more than three metastatic sites. Overall, 148 patients (83.6%) received the treatment in the first line and 29 (16.4%) in the second line. One hundred forty‐four patients (81.3%) received D/T, 29 V/C (16.4%), and only 4 (2.3%) were treated with E/B. Seventy‐eight patients (44.8%) were assigned to the Risk‐H category, while 99 patients (55.9%) to the clinical Risk‐L one.

**TABLE 1:** Clinical‐pathological features.

Characteristics	All patients *N* 177 (%)
Age—years	
Median age (range)	62 (23–88)
Gender	
Male	117 (66.1)
Female	60 (33.9)
Performance status	
0	103 (58.2)
1	55 (31.1)
2	19 (10.7)
Comorbidity	
Yes	118 (66.6)
No	59 (33.4)
Line of treatment anti‐BRAF	
I	148 (83.6)
II	28 (15.8)
III	1 (0.6)
Metastatic sites	
< 3	105 (59.3)
≥ 3	72 (40.7)
Site of metastasis at baseline	
Liver	83 (46.9)
Lung	90 (50.8)
Lymph nodes	99 (55.9)
Bone	34 (19.2)
Skin	32 (18.0)
Brain	44 (24.8)
Soft tissue	50 (28.2)
Locoregional recurrence	6 (3.4)
Pleura	3 (2.2)
Peritoneum	14 (7.9)
Type of anti‐BRAF therapy	
Dabrafenib/trametinib	144 (81.3)
Vemurafenib/cobimetinib	29 (16.4)
Encorafenib/binimetinib	4 (2.3)
Risk category	
High	78 (44.8)
Low	99 (55.9)

### 3.2 Concomitant Medications and the Degree of Drug–Drug Interactions

In 118 patients, at least one comorbidity was recorded. Ninety‐nine patients (55.9%) required specific pharmacological treatment for comorbidities, supportive care, or symptom control, leading to the assumption of at least one concomitant medication before starting anti‐BRAF/MEK inhibitors (Table S1). 22 patients took five or more daily drugs.

At the baseline, the Drug‐PIN light was green in 157 patients (88%), yellow in 10 (6%), dark yellow, and red in 10 patients (6%) (comprising 9 and 1 patients, respectively). When the combined BRAF‐i/MEK‐i treatment was added to home therapy (Table S2), the Drug‐PIN light turned green in 112 patients (63%), yellow in 28 (16%), and dark yellow or red in 37 (21%) patients (22 and 15 patients, respectively). A statistically significant difference was evidenced between pre‐ and post‐therapy Drug‐PIN light (Wilcoxon rank test *p*‐value < 0.001).

Therefore, 167 and 140 patients were classified as DDI‐L, while 10 and 37 were categorized as DDI‐H before and following BRAF‐i/MEK‐i treatment.

### 3.3 Outcomes: Best Response, PFS, and OS According to Clinical Features

**FIGURE 1:**
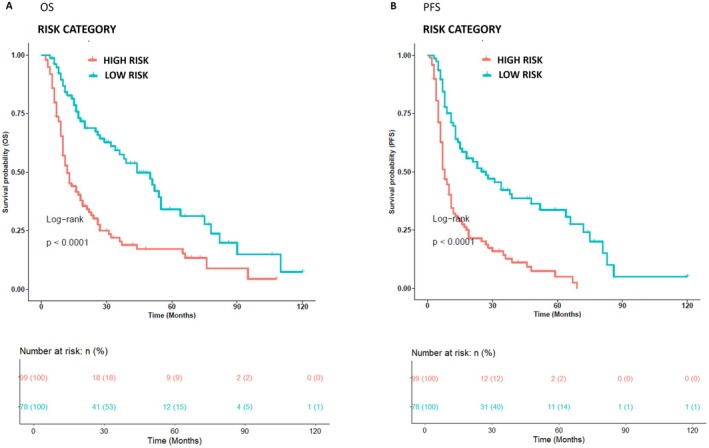
Kaplan–Meier analysis for clinical risk classes (Risk‐H vs. Risk‐L). 177 patients were classified into two groups: one class including clinical Risk‐H (99 patients, red curve) and the other one including Risk‐L patients (78 patients, cyan curve). The correlation between variable value and patient survival was examined as OS [panel A] and PFS [panel B]. The prognosis of each group of patients was analyzed by Kaplan–Meier survival estimators, and the survival outcomes of the two groups were compared by log‐rank tests. Log‐rank *p*‐values less than or equal to 0.05 were considered statistically significant. Patients in the Risk‐L class show a better outcome than those in the Risk‐H class in terms of OS and PFS.

The oncological outcomes of best response, PFS, and OS are shown in Table 2. In the overall study population, PD, SD, PR, and CR occurred in 23 (20%), 38 (21.5%), 98 (55.4%), and 18 (10.1%) patients, respectively. Sixteen (16.1%) patients reported PD in the clinical Risk‐H group, while 22 (22.2%), 58 (58.6%), and 3 (3%) patients achieved SD, PR, and CR, respectively. In 7 patients (9%) with the Risk‐L group, PD occurred, while SD, PR, and CR occurred in 16 (20.5%), 40 (51.3%), and 15 (19.2%) patients, respectively. The median OS in the study population was 19 months (2–120). As expected, patients belonging to the Risk‐H group had a significantly worse OS and PFS than patients belonging to the Risk‐L one (*p* < 0.0001 and *p* < 0.0001, respectively); in the Risk‐H group, the median OS was 11 months (2–108) versus 30.5 (4–120) in the Risk‐L group (HR = 2.41, 95% CI (1.7–3.5), *p*‐value < 0.0001). Similarly, the median PFS was 7 months (1–79) and 20 months (3–120) in the Risk‐H and Risk‐L groups, respectively (HR = 2.72, 95% CI (1.9–3.9), *p*‐value < 0.0001) (Figure 1A/B).

**TABLE 2:** Outcomes: Best response, OS, and PFS in the overall study population and according to DDIs and risk subgroups (high and low).

Parameter	Patients *N* (%)	DDI‐L (%)	DDI‐H (%)	Clinical RISK‐H (%)	Clinical RISK‐L (%)
Patients	177 (100)	140 (79)	37 (21)	99 (56)	78 (44)
Best response					
Progressive disease	23 (20.0)	13 (9.3)	10 (27.0)	16 (16.1)	7 (9.0)
Stable disease	38 (21.5)	30 (21.4)	8 (21.6)	22 (22.2)	16 (20.5)
Partial response	98 (55.4)	81 (57.8)	17 (45.9)	58 (58.6)	40 (51.3)
Complete response	18 (10.1)	16 (11.4)	2 (5.4)	3 (3)	15 (19.2)

Survival	Median (range) months				
OS	19 (2–120)	19 (3–120)	11 (2–82)	11 (2–108)	30.5 (4–120)
PFS	13 (2–120)	12 (2–120)	7 (2–81)	7 (1–79)	20 (3–120)

Abbreviations: DDI, drug–drug interactions; OS, overall survival; PFS, progression‐free survival.

Moreover, responder patients had significantly better OS and PFS than non‐responder patients (Figure S1A/B). Furthermore, the best response to treatment significantly impacted OS and PFS (*p* < 0.0001), with the best outcome reported by patients achieving complete response (Figure S2).

### 3.4 Associations of Outcomes and Drug‐PIN

During combo target treatment, PD occurred in 13 (9.3%) and 10 (27%) patients with DDI‐L and DDI‐H, respectively. Thirty (21.4%), 81 (36.8%), and 16 (11.4%) patients with DDI‐L reported SD, PR, and CR, respectively. In patients with DDI‐H, SD, PR, and CR occurred in 8 (21.6%), 17 (45.9%), and 2 (5.4%) patients, respectively.

**FIGURE 2:**
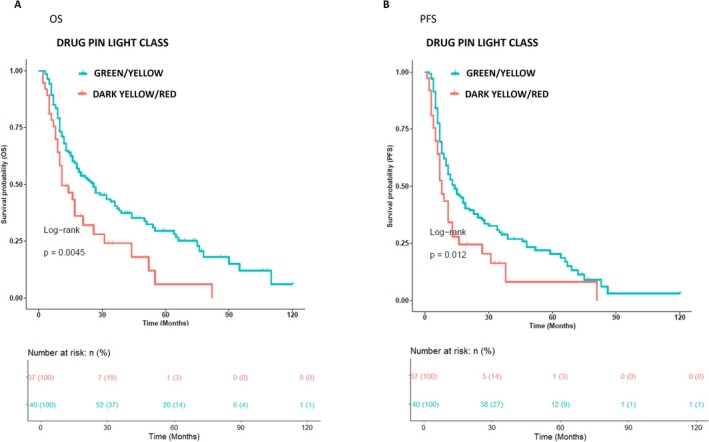
Kaplan–Meier analysis on DDI for all patient cohorts. 177 patients were classified into two groups: one class, including patients with DDI‐L (37 patients, red curve), and the other one, including patients with DDI‐L (140 patients, cyan curve). The correlation between variable value and patient survival was examined as OS [panel A] and PFS [panel B]. The prognosis of each group of patients was analyzed by Kaplan–Meier survival estimators, and the survival outcomes of the two groups were compared by log‐rank tests. Log‐rank *p*‐values less than or equal to 0.05 were considered statistically significant. Patients with DDI‐L showed a good prognosis compared to those with DDI‐H classes in terms of OS and PFS.

Patients with DDI‐L had a significantly better median OS and PFS than patients with DDI‐H (log‐rank *p*‐value = 0.0045 and *p*‐value = 0.012, respectively), as shown in Figure 2A/B. Median OS was 19 months (3–120) and 11 months (2–82) in DDI‐L and DDI‐H, respectively (HR = 1.83, 95% CI (1.2–2.8), *p*‐value = 0.005), and median PFS was 12 (2–120) and 7 months (1–81) in DDI‐L and DDI‐H, respectively (HR = 1.68, 95% CI (1.1–2.5), *p*‐value = 0.0128) (Figure 2).

Considering patients according to treatment response, neither responder nor non‐responder patients showed a statistically significant association between DDI‐L or DDI‐H and both OS and PFS, as shown in Figures S3A/B and S4A/B. A trend toward significance in DDI‐L responder patients with a better OS (*p* = 0.085, Figure S4A) was evidenced. Median OS was 22 months (3–120) in DDI‐L/responder patients versus 13 months (3–82) in DDI‐H/responder patients (HR = 1.55, 95% CI (0.94–2.6), *p* = 0.085). However, median PFS was 13 months (3–120) and 11 months (3–81) in DDI‐L and DDI‐H/responder patients, respectively (HR = 1.37, 95% CI (0.84–2.2), *p*‐value = 0.20).

### 3.5 Association of Outcome, Clinical Risk, and DDI

Considering the 99 patients in the Risk‐H group, DDI was statistically significantly associated with OS (*p* = 0.031). Risk‐H/DDI‐L patients had a significantly better OS than those with Risk‐H/DDI‐H 12 months (3–108) versus 9.5 months (2–83), respectively, (HR = 1.79, 95% CI (1–3.1), *p*‐value = 0.0346). Regarding PFS, patients with Risk‐H/DDI‐L have a better PFS than those with Risk‐H/DDI‐H, although this finding does not reach statistical significance. Median PFS was 6.5 (1–33) in Risk‐H/DDI‐L patients versus 8 months (2–69) in Risk‐H/DDI‐H patients (HR = 1.58, 95% CI (0.93–3.7), *p*‐value = 0.089).

**FIGURE 3:**
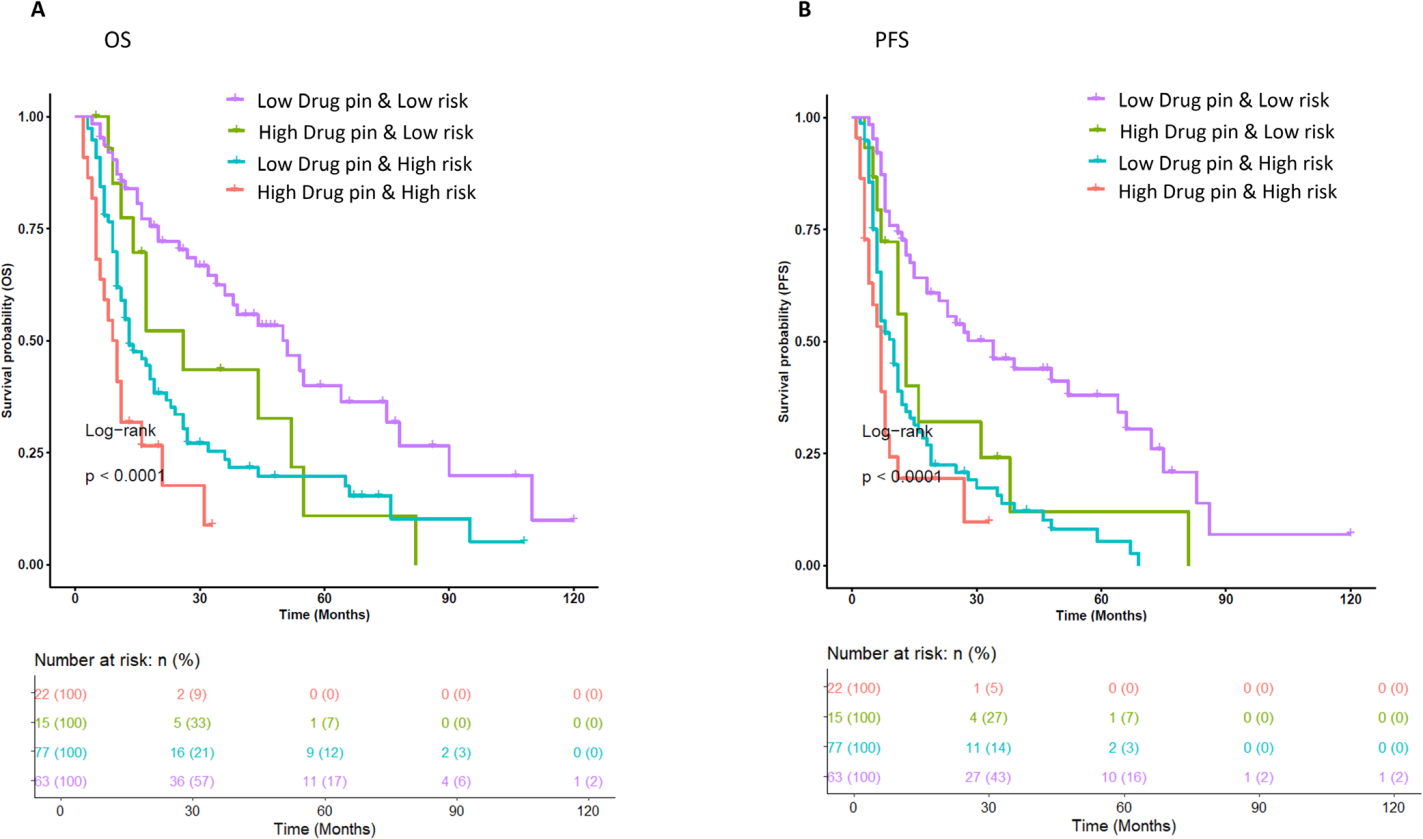
Kaplan–Meier analysis on Drug‐PIN light and risk patient status. 177 patients were classified into 4 groups: one class including patients with Risk‐H/DDI‐H (22 patients, red curve), one including patients with Risk‐L/DDI‐H (15 patients, green curve), one including patients with Risk‐H/DDI‐L (77 patients, cyan curve), and finally one including patients with Risk‐L/DDI‐L (63 patients, violet curve). The correlation between variable value and patient survival was examined as OS [panel A] and PFS [panel B]. The prognosis of each group of patients was analyzed by Kaplan–Meier survival estimators, and the survival outcomes of the two groups were compared by log‐rank tests. Log‐rank *p*‐values less than or equal to 0.05 were considered statistically significant. Patients in Risk‐H and DDI‐H classes showed a worse prognosis than those in Risk‐L and DDI‐L classes in terms of OS and PFS.

Regarding the 78 patients in the Risk‐L category, DDI was not statistically significantly associated with either OS (*p* = 0.062) or PFS (*p* = 0.2). However, there was a trend toward statistical significance in OS that tended to be better in patients with Risk‐L/DDI‐L than those with Risk‐L/DDI‐H, as shown in Figure 3A, 17 months (5–82) and 32 months (4–120), respectively (HR = 1.91, 95% CI (1–3.8), *p*‐value = 0.063). On the other hand, median PFS was 11 months (3–81) and 25 months (4–120) in Risk‐L/DDI‐H and Risk‐L/DDI‐L, respectively (HR = 1.98, 95% CI (1–3.8) *p*‐value = 0.041) (Figure S5).

Furthermore, four patient groups were established based on the DDI and the risk category. For each group, a survival analysis was performed in terms of OS and PFS. Comparing the 4 patient groups, a statistically significant difference in terms of both OS and PFS was confirmed (log‐rank *p*‐value < 0.0001), as shown in Figure 3A/B. Median OS was 32 (5–82), 12 (2–33), 17 (5–82), and 9.5 (2–33) months in the Risk‐L/DDI‐L, Risk‐H/DDI‐L, Risk‐L/DDI‐H, and Risk‐H/DDI‐H groups, respectively.

The Risk‐L/DDI‐L group, composed of 63 patients, had the best OS compared to the other groups (HR = 0.39, 95% CI (0.26–0.58), *p*‐value < 0.001), followed by Risk‐L/DDI‐H (15 patients) (HR = 1.03, 95% CI (0.55–1.9), *p*‐value = 0.931) and Risk‐H/DDI‐L (77 patients) (HR = 1.66, 95% CI (1.2–2.4), *p*‐value 0.005) (Table 3). On the other hand, the Risk‐H/DDI‐H group had the worst OS (HR 2.87, 95% CI (1.7–4.8), *p*‐value < 0.001). The Risk‐L/DDI‐L group also eported the best PFS (25 months (3–81), HR = 0.353, 95% CI (0.24–0.52), *p*‐value < 0.0001), compared to other groups: Risk‐L/DDI‐H (11 months (3–81), HR = 1.03, 95% CI (0.57–1.9), *p*‐value = 0.910), Risk‐H/DDI‐L (8 months (1–33), HR = 1.96, 95% CI (1.4–2.8), *p*‐value = 0.0001), and the Risk‐H/DDI‐H group (6.5 months (1–33), HR = 2.47, 95% CI (1.5–4.1), *p*‐value < 0.0001) (Table 3).

**TABLE 3:** OS and PFS analysis based on Drug‐PIN light and risk patient status.

Group	OS, months (range)	HR (95% CI)	*p*	PFS, months (range)	HR (95% CI)	*p*
Risk‐H/DDI‐H	9.5 (2–33)	HR 2.87, 95% CI (1.7–4.8)	< 0.001	6.5 (1–33)	HR = 2.47, 95% CI (1.5–4.1)	< 0.0001
Risk‐L/DD‐L	32 (5–82)	HR = 0.39, 95% CI (0.26–0.58)	< 0.001	25 (3–81)	HR = 0.353, 95% CI (0.24–0.52)	< 0.0001
Risk‐L/DDI‐H	17 (5–82)	HR = 1.03, 95% CI (0.55–1.9)	0.931	11 (3–81)	HR = 1.03, 95% CI (0.57–1.9)	0.910
Risk‐H/DDI‐L	12 (2–33)	HR = 1.66, 95% CI (1.2–2.4)	0.005	8 (1–33)	HR = 1.96, 95% CI (1.4–2.8)	0.0001

### 3.6 Multiple Regression Analysis for Response and Survival

Cox multiple regression model was performed to test which variables were associated with OS and PFS. Considering age, sex, number of metastatic sites ≥ 3, ECOG PS, DDI‐L or DDI‐H, and treatment line, a statistically significant association was found between sex, number of metastatic sites, DDI, ECOG‐PS, and OS. Moreover, female gender was also significantly associated with better OS (*p* = 0.01). DDI at multiple regression analysis had a higher significance (*p*‐value = 0.0019) than sex (*p*‐value = 0.01). At the same time, the factors most significantly predictive of OS were the number of metastasis sites (*p*‐value = 1.35E‐05) and ECOG‐PS (0.0008), as shown in Table 4. Gender, number of metastatic sites, DDI, and ECOG PS were also significantly associated with PFS. In particular, the number of metastatic sites (*p*‐value = 2.77E‐06), followed by Drug‐PIN light (*p*‐value = 0.008) and ECOG‐PS (*p*‐value = 0.004), were the most significant variables associated with PFS at multiple regression analysis, as shown in Table 5.

**TABLE 4:** Multiple regression analysis: Association between different clinical‐pathological characteristics and OS.

Variables—OS	Beta	Hazard ratio (95% CI for HR)	*p*
Age	−0.00339	0.997 (0.98–1)	0.63
Gender (male vs. female)	−0.5149	0.598 (0.4–0.88)	0.01**
Metastasis	0.813555	2.26 (1.6–3.3)	1.35E‐05***
DDI	0.179597	1.28(1–1.4)	0.002**
ECOG‐PS	0.638439	1.89 (1.3–2.7)	0.0008***
Therapy line	−0.43261	0.649 (0.4–1.1)	0.09

Abbreviations: DDI, drug–drug interactions; ECOG PS, Eastern Cooperative Oncology Group performance status; OS, overall survival.

** *p* ≤ 0.01.

*** *p* ≤ 0.001.

**TABLE 5:** Multiple regression analysis: Association between different clinical‐pathological characteristics and PFS.

Variables—PFS	Beta	Hazard ratio (95% CI for HR)	*p*
Age	−0.00206	0.998 (0.98–1)	0.76
Gender (male vs. female)	−0.39256	0.675 (0.47–0.97)	0.03*
Metastasis	0.856635	2.36 (1.6–3.4)	2.77E‐06***
DDIs	0.19899	1.22 (1.1–1.4)	0.008**
Performance status (ECOG PS)	0.506927	1.66 (1.2–2.3)	0.004**
Therapy line	−0.41507	0.66 (0.42–1)	0.08

Abbreviations: DDI, drug–drug interactions; ECOG PS, Eastern Cooperative Oncology Group performance status; PFS, progression free survival.

* *p* ≤ 0.05.

** *p* ≤ 0.01.

*** *p* ≤ 0.001.

### 3.7 Drugs Associated With a High Degree of Interaction (DDI‐H) With BRAF‐i and MEK‐i

The 37 patients with DDI‐H were assessed. In the 37 patients with significant pharmacological interactions, the most frequently co‐administered drugs were: Antihypertensives (12 patients were on beta‐blockers, 11 patients ACE inhibitors; 11 patients were using diuretics, 11 patients were on calcium antagonists, 9 patients were taking angiotensin receptor blockers (ARBs)), Psychoactive medications (8 patients were using benzodiazepines; 5 patients were taking selective serotonin reuptake inhibitors (SSRIs) or other antidepressants; and 3 patients were on antipsychotics), Antacids (11 patients were using proton pump inhibitors and H2‐antagonists), Antiplatelets (9 patients were on acetylsalicylic acid at anti‐platelet dosage and 3 patient was taking clopidogrel), Lipid‐lowering agents (9 patients were on statins and/or other oral hypo‐lipidemics), Antidiabetics (2 patients were using insulin; 6 patients were taking oral hypoglycemics), Medications for benign prostatic hyperplasia (5 patients were on alpha‐adrenergic antagonists), Antiepileptics (4 patients), Antiasthmatics (4 patients), Antigout medications (4 patients were taking allopurinol and/or febuxostat), Anticoagulants: 4 patients were on warfarin or other direct oral anticoagulants.

Less frequently represented among the sample were the following medications: antiarrhythmics (3 patients), bile acids (2 patients), antianginal medications (1 patient), synthetic thyroid hormones (2 patients), medications for myasthenia gravis (1 patient on acetylcholinesterase inhibitors), analgesics/anti‐inflammatories (4 patients on oxycodone, 4 patients on steroids), and antiandrogens and GnRH analogs (2 patients).

The classes of medications demonstrating a high and low risk of DDIs in conjunction with BRA‐i and MEK‐i, along with their pharmacodynamic and pharmacokinetic modulation, are presented in Tables 6 and 7, respectively. An additional table has been created to specify the individual molecules recommended for avoidance or preference based on their interaction effects (Table 8).

**TABLE 6:** High‐risk DDI drugs potentially interacting with BRAF/MEK inhibitors.

Class	Drug	High‐risk association	Mechanism	Effect
Antihypertensive	Amlodipine, Nifedipine	Dabrafenib	CYP3A4 inhibitors	May alter systemic exposure to dabrafenib
	Losartan	Vemurafenib Cobimetinib Amiodarone Valproate	Conversion to active metabolite EXP‐3174 through CYP2C9 e CYP3A4	Reduced antihypertensive activity of losartan
	Carvedilol	Dabrafenib (inducer of CYP2C9)	Substrate of CYP2D6 and CYP2C9	Reduced exposure to carvedilol
	Bisoprolol	Dabrafenib trametinib Warfarin (inducers of CYP3A4/5/7) Spironolactone (inducer of PGP)	Substrate of CYP3A4/5 and PGP	Reduced systemic exposure to bisoprolol when co‐administered with dabrafenib and trametinib
Antiarrhythmics	Amiodarone	Dabrafenib (Inducer of CYPD9)	Potent inhibitor of CYP3A4, CYP2C9, and PGP	Reduced systemic exposure to amiodarone and increased systemic exposure to dabrafenib
	Dronedarone	Dabrafenib	Inhibitor of CYP3A4 and PGP	Increased systemic exposure to dabrafenib
	Flecainide	Vemurafenib (inhibitor of CYP1A2)	Substrate of CYP1A2	Increased systemic exposure to flecainide and prolongationof QT interval
	Class IA, Ic, and III antiarrhythmics	Vemurafenib	CYP1A2	*Prolongation QT interval*
Hypolipidemic drugs	Lovastatin, simvastatin, and atorvastatin	Dabrafenib Vemurafenib Encorafenib Dihydropyridine calcium antagonists	Inhibitors of CYP3A4/5/7	Increased systemic exposure to dabrafenib, vemurafenib, and encorafenib
Anticoagulants	Warfarin	Dabrafenib (inducer of CYP2C9)	Substrate of CYP2C9	Reduced systemic exposure to warfarin
	Rivaroxaban, Apixaban	Dabrafenib Vemurafenib Cobimetinib (inducers of CYP3A4)	Substrate of CYP3A4 (*dabrafenib*, *V/C*)	Reduced systemic exposure to direct oral anticoagulant agent
Antiplatelet	ASA	Vemurafenib (inhibitor of CYP1A2, 2C8, 2C9, and 2D6) Cobimetinib (inhibitor of CYP2C8, CYP2D6, CYP3A4/5/7)	ASA is metabolized by CYP2C8/9	Increased systemic exposure to ASA, increased risk of bleeding
Anti‐reflux drugs	Proton pump inhibitors	Dabrafenib Vemurafenib Encorafenib Benzodiazepine	Inhibitors of CYP3A4	Increased systemic exposure to dabrafenib, vemurafenib, and encorafenib
Anti‐epileptic drugs	Carbamazepine	Dabrafenib	Inducer of CYP3A4, PGP	Reduced systemic exposure to dabrafenib
Benzodiazepine	Alprazolam	Dabrafenib (inducer of CYP3A4)	Substrates of CYP3A4	Increased systemic exposure to alprazolam and dabrafenib
Antidepressants	Citalopram	Dabrafenib (inducer of CYP3A4)	Substrate of CYP3A4	Increased systemic exposure to citalopram and dabrafenib
	Fluoxetine, Fluvoxamine, Paroxetine, Sertraline, Duloxetine, Venlafaxine, Mirtazapine	Vemurafenib Cobimetinib (inhibitors of CYP2C8/9 e CYP2D6)	Competition on CYP2D6	Increased systemic exposure to SSRI

Abbreviation: SSRI, selective serotonin reuptake inhibitors. ASA, acetylsalicylic acid.

**TABLE 7:** Low‐risk DDI drugs in combination with BRAF/MEK inhibitors.

Class	Drugs	Pharmacokinetics
Antihypertensives	ACE inhibitors	The Drug‐PIN score was not significantly elevated by the addition of ACE inhibitors. This category of drugs is predominantly renally eliminated. They do not alter liver cytochrome function to such an extent that systemic exposure to BRAF/MEK inhibitors is altered
	Olmesartan Valsartan Candesartan	It is not a substrate of any enzyme involved in the metabolism of BRAF/MEK inhibitors
	Clevidipine	Minimal involvement of cytochromes
	Nebivololo	Metabolized via CYP2D6
Antiarrhythmics	Dofetilide	Minimum CYP involvement
Hypolipidemic drugs	Pravastatin	Biliary and urinary excretion metabolism
Anticoagulants	Enoxaparin	Renal metabolism
	unfractionated heparin	Renal metabolism
Antireflux drugs	Sodium Alginate	Fecal excretion
	Magaldrate	Fecal excretion
Antiepileptics	Levetiracetam	Hydrolysis (hepatic amidases) Renal excretion
	Oxcarbazepine, Eslicarbazepine	Non‐CYP‐mediated activation and glucurono‐conjugation Renal excretion
Benzodiazepine	Delorazepam	Minimal CYP involvement Urinary excretion
	Lorazepam	Glucurono‐conjugation Urinary excretion
Oral Antidiabetics	Metformin	Renal tubular secretion
Opioid drugs	Oxycodone, fentanyl	Although metabolized by cytochromes, they are not associated with a high risk of interaction with BRAF/MEK inhibitors
	Morphine, tramadol, codeine	Metabolized mainly by glucuronidation in the liver
Non‐steroidal anti‐inflammatory drugs	Ketorolac, ibuprofen	Metabolized mainly by glucurono‐conjugation
Corticosteroids	Hydrocortisone, Prednisone, Dexamethasone, Methylprednisolone, Betamethasone	Reduced systemic exposure to corticosteroids

**TABLE 8:** Recommendations for drugs to avoid or prefer based on their potential pharmacological interactions with BRAF and MEK inhibitors.

Class	Recommended	Not recommended
ACE inhibitors	All drugs in the category are recommended	—
Sartans	Olmesartan, Valsartan, Candesartan	Losartan
Calcium antagonist	Clevidipine	Amlodipine, Nifedipine
Beta‐blockers	Nebivolol	Carvedilol, Bisoprolol
Antiarrhythmics	Dofetilide	Amiodarone, Dronaderone, Flecainide
Hypolipidemic drugs	Pravastatin	Lovastatin, simvastatin, and atorvastatin
Anticoagulants	Enoxaparin Unfractionated heparin	Warfarin, Rivaroxaban, Apixaban
Anti‐platelets	Clopidogrel	ASA
Antireflux drugs	Sodium Alginate Magaldrate	Proton pump inhibitors
Antiepileptics	Levetiracetam Oxcarbazepine, Eslicarbazepine	Carbamazepine
Benzodiazepine	Delorazepam Lorazepam	Alprazolam
Antidepressants	Fluvoxamine, paroxetine, sertraline, mirtazapine, and duloxetine have a low potential for interaction with dabrafenib and trametinib but a high risk of interaction with vemurafenib/cobimetinib	Citalopram
Alpha‐blockers	Prazosin	Alfuzosine and tamsulosine (During treatment with BRAF/MEK inhibitors the use of a double alpha blocker is to be avoided)
Oral antidiabetics	Metformin	—
Opioid drugs	All drugs recommended^a^	—
Non‐steroidal anti‐inflammatory drugs	Recommended (ketorolac, ibuprofen)	—
Corticosteroids	Hydrocortisone, Prednisone, Dexamethasone, Methylprednisolone, Betamethasone^b^	—

Abbreviation: ASA, acetylsalicylic acid.

^a^ The oxycodone‐naloxone combination, although associated with a potentially low risk of DDIs, results in a Drug‐PIN score of light yellow, indicating that it should be used with caution in cases of polypharmacotherapy.

^b^ Moderate interactions with dabrafenib.

## 4 Discussion

This study highlights that in patients with BRAF^v600^ MM treated with BRAF‐i/MEK‐i, the presence of DDI‐H emerges as a negative prognostic factor; in particular, DDI appears to be significantly associated with worse oncological outcomes in terms of OS and PFS regardless of clinical risk. Drug interactions provide a relevant issue in the population studied. Approximately 56% of patients were exposed to complex drug regimens (related to comorbidities, supportive care, and symptom control) before starting cancer treatment. Potentially dangerous DDIs were identified in 10 patients before starting therapy with BRAF‐i/MEK‐i. The addition of BRAF‐i/MEK‐i treatment significantly increased the DDI score, which was associated with a relevant change in Drug‐PIN light in 54 patients. In our population, the DDI‐L class consisted mainly of patients with a green color range but also included a limited number of patients with medium drug interactions (Drug‐PIN score = 20–30). The DDI‐H class included patients with Drug‐PIN light from dark yellow to red, with a Drug‐PIN score > 30, in which the tool demonstrated a high risk of DDI drug cocktail (Borro et al. 2021). In some cases, we have identified therapies that include drugs with significant interactions with BRAF/MEK inhibitors, which justifies the high degree of DDIs; in other instances, the combination of multiple agents competing for the same enzyme or having inhibition/induction activity on the same enzyme was responsible for the elevated DDI score. This analysis identifies several contraindicated drugs for co‐administration with BRAF‐i/MEK‐i, particularly amlodipine and nifedipine, amiodarone and dronedarone, lipid‐lowering agents such as atorvastatin, lovastatin, and simvastatin, as well as proton pump inhibitors, due to their inhibitory activity on CYP3A4. Special caution must be exercised when administering molecules that prolong the QT interval, such as antiarrhythmics, antipsychotics, tricyclic antidepressants, and antiemetics. The International Normalized Ratio (INR) must be closely monitored in patients treated with warfarin and BRAF/MEK inhibitors. Co‐administration of benzodiazepines with BRAF‐i/MEK‐i and other CYP3A4 substrates should be avoided. Conversely, ACE inhibitors, olmesartan, clevidipine, dofetilide, pravastatin, enoxaparin, metformin, and certain antiepileptic drugs such as levetiracetam, oxcarbazepine, eslicarbazepine, opioids, and corticosteroids can be administered without significant interactions due to minimal involvement of cytochromes in their metabolism. This work highlights how, through the Drug‐PIN software, it is possible to optimize ongoing treatments for non‐oncological comorbidities to reduce the risk of DDIs in a population of patients receiving combination targeted therapy with BRAF‐i/MEK‐i. Given the sample size, it is deemed useful to plan a prospective analysis in a larger population to confirm or refute the observations, especially concerning the DDIs of the BRAF‐i/MEK‐i vemurafenib/cobimetinib and encorafenib/binimetinib.

Particularly, our analysis highlights that DDI‐H confers a negative prognostic impact on MM patients receiving treatment with BRAF‐i/MEK‐i, highlighted by the highest progressive disease rate. These data on outcomes deterioration show, according to Drug‐PIN, a significantly worse OS and PFS in patients with DD‐H than in patients with DDI‐L (*p* = 0.0045 and *p* = 0.012, respectively). Furthermore, the critical role of DDI on outcomes was confirmed by multiple regression analysis, which also demonstrated the predictive role of Drug‐PIN light and score concerning the prognosis deterioration in MM patients compared to several other established clinical parameters (age, gender, ECOG‐PS, and number of metastatic sites).

In line with the literature, aggressive clinical features significantly impact OS and PFS^373839^. Moreover, combining clinical and DDI features, we identified four classes of patients with different outcomes. Comparing these 4 patient groups, a statistically significant difference in both OS and PFS was confirmed (log‐rank *p*‐value < 0.0001). In particular, clinical Risk‐H/DDI‐H patients had a significantly worse OS than those with Risk‐H/DDI‐L (12 months (3–108) versus 9.5 months), allowing us to identify the Risk‐H/DDI‐H as the group of patients with the worst prognosis (HR 2.87, 95% CI (1.7–4.8), *p*‐value < 0.001).

Even if correlation does not imply a cause‐effect relation, we can assume that DDIs have a negative effect within the same prognostic group with a reduction in median OS of 2.5 and 15 months, respectively, and in PFS of 1.5 and 14 months, in the Risk‐H and Risk‐L classes, indirectly confirming the harmful effect of DDIs on the outcome. This significant effect on outcomes may depend on pharmacokinetic modulation or, indirectly, on the frailty of patients with comorbidities. Additionally, a multitude of concomitant factors can substantially influence patient outcomes. The concurrent use of multiple medications, which increases the risk of DDIs, particularly underscores a vulnerable population characterized by advanced age, comorbidities, progressive disease, and symptomatic manifestations of their advanced condition, all of which adversely impact patient outcomes (Borro et al. 2021).

The Drug‐PIN analysis incorporates several critical factors, including age‐related pharmacokinetics, nutritional status, pharmacogenomic variations, and biochemical profiles. However, the current version of the Drug‐PIN system has notable limitations due to its failure to integrate the QT interval and serum albumin concentration as evaluative parameters. One significant shortcoming is the lack of QT interval analysis. The system does not dynamically assess the risk of QT prolongation, which is crucial for many medications with proarrhythmic potential. Similarly, the absence of serum albumin consideration represents another critical flaw. Serum albumin levels play a key role in the pharmacokinetics of highly protein‐bound medications, such as warfarin and diazepam. Low serum albumin levels—commonly observed in frail or critically ill patients—can lead to increased free drug concentrations, thereby elevating the risk of toxicity. Without accounting for this parameter, the system may underestimate the likelihood of adverse drug reactions in vulnerable patient populations. Moreover, the influence of comorbidities on treatment complexity, medication adherence, access to healthcare, and side effects can only be assessed indirectly, contributing to the variability often observed in clinical outcomes. DDIs usually involve the cytochromes of the P450 (CYPs) enzyme superfamily, which are also involved in BRAF‐i/MEK‐i drug metabolism. DDIs may influence BRAF‐i/MEK‐i plasma concentrations and drug activity, probably affecting both treatment efficacy and adherence to treatment (Borro et al. 2021). In MM patients treated with BRAF‐i/MEK‐i, DDI plays a prognostic role, as DDIs are associated with worse oncological outcomes. Therefore, all patients who must start treatment with BRAF and MEK inhibitors should be evaluated for potential DDIs due to the possible implications in worsening prognosis in advanced disease and potentially in the adjuvant setting. To mitigate the confounding effects of DDIs on patient outcomes, implementing effective strategies is paramount. Conducting regular medication reviews, maintaining vigilant monitoring for potential DDIs, and utilizing comprehensive screening tools can empower healthcare providers to identify and manage these interactions efficiently, ultimately leading to more personalized and safer treatment approaches.

The primary limitation of this study is its retrospective design, which does not allow for a more in‐depth investigation of every aspect of drug interactions. Indeed, these results suggest that the Drug‐PIN score and light may have an important prognostic value for oncological outcomes, which should be further studied in prospective and targeted studies, leading to the optimization of therapy based on the assessment of potential DDIs and the use of alternative medications when feasible.

## 5 Conclusions

Polytherapies in oncological patients significantly increase the frequency of unfavorable DDIs.

This study provides several essential indications regarding the impact of DDIs on the outcome of BRAF^v600^ MM patients treated with combined BRAF‐i/MEK‐i, offering insight into drug–drug interactions in a *real‐world* population. Therefore, optimizing the pharmacological treatments taken by each patient is essential, given the detrimental impact on the oncological outcomes of DDIs. A prospective clinical trial to confirm the added value in terms of outcomes of individualized preemptive therapy optimization is needed.

## Disclosure

Institutional Review Board Statement: The studies involving humans were approved by Sapienza University of Rome Prot. 0435/2021 Rif. 6332. The studies were conducted in accordance with local legislation and institutional requirements.

## Consent

The participants provided written informed consent to participate in this study. No animal studies are presented in this manuscript. No potentially identifiable images or data are shown in this study.

## Conflicts of Interest

Silvia Mezi (S.M.) speaker fee for Novartis, Pierre‐Fabre, BMS, and MSD; Andrea Botticelli (A.B.) speaker and advisory board for BMS, Novartis, Pfizer, Lilly, MSD, Daiichi‐Sankyo, and Gilead; Simone Scagnoli (S.S.) has/had consulting fees with Pfizer, Novartis, and Lilly; Federica De Galitiis has/had consulting fees with Novartis, BMS, MSD, and Pierre‐Fabre; Simona Pisegna (S.P.) speaker fee for Novartis, Roche, Pfizer, AstraZeneca, and Lilly; Maurizio Simmaco (M.S.) is a member of the Advisory Board of Drug‐PIN AG; Paolo Marchetti (P.M.) consultant/advisory role for BMS, Roche Genentech, MSD, Novartis, Amgen, Merck Serono, Pierre Fabre, and Incyte. Paolo Marchetti, Maurizio Simmaco, and Robert Preissner are members of the advisory board of Drug‐PIN AG (software cited in the text). The Drug‐PIN AG is the holder of patent PCT/IB2019/052310. The remaining authors declare to have no conflicts of interest.

## Supporting information


Appendix S1.

Appendix S2.

## Data Availability

Individual participant data that underlie the results reported in this article, after de‐identification, study protocol, informed consent form, and statistical analysis plan will be available immediately following publication, with no end date, to researchers who provide a methodologically sound proposal to achieve aims in the approved proposal. Proposals should be directed to simone.scagnoli@uniroma1.it to gain access; data requestors must sign a data access agreement.
