# Supplementary material for: Impact of Drug–Drug Interactions on Clinical Outcomes in Metastatic Melanoma Patients Treated With Combined BRAF/MEK Inhibitors: A Real‐World Study
Source: Pigment Cell Melanoma Res. 2025 Jun 1;38(4):e70026. doi: 10.1111/pcmr.70026 (PMC12127241; doi:10.1111/pcmr.70026)
Supplement: Supplementary file 1 — Appendix S1. [file PCMR-38-0-s002.docx]

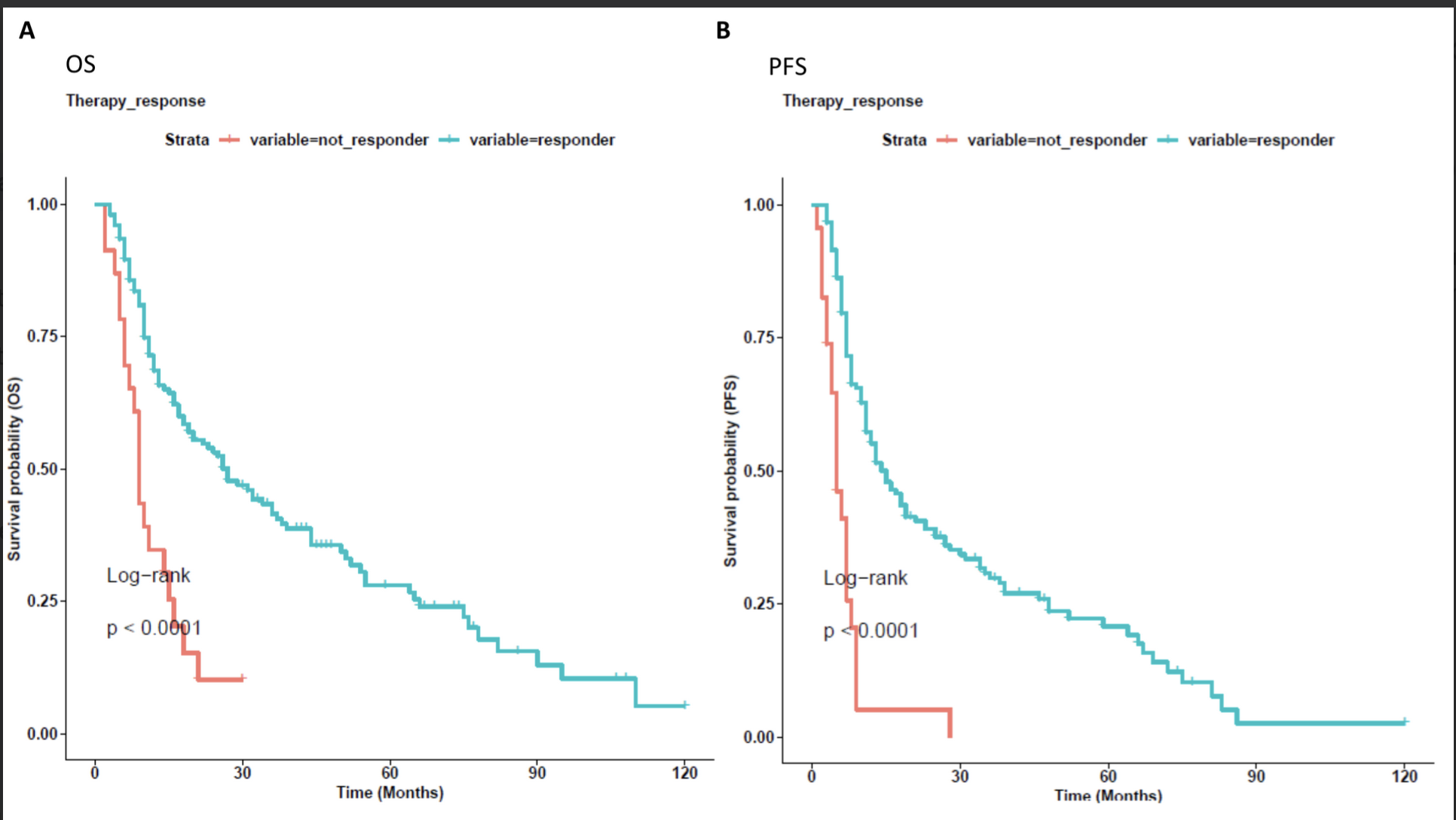


**Figure S1: Trends in Outcomes Based on Treatment Response to BRAF-MEK Inhibitors in All Patients**. **Kaplan-Meier analysis.**

177 patients were classified into two groups: one class including not responder patients (23 patients, red curve), and the other one including responder patients (154 patients, cyan curve). The correlation between variable value and patient survival was examined as overall survival (OS) [panel A] and progression free survival (PFS) [panel B]. The prognosis of each group of patients was examined by Kaplan-Meier survival estimators, and the survival outcomes of the two groups were compared by log-rank tests. Log rank p-values less than or equal to 0.05 were considered as statistically significant. Patients belonging responder class show a good prognosis with respect to the ones belonging to not responder class both in terms of OS and PFS.

**
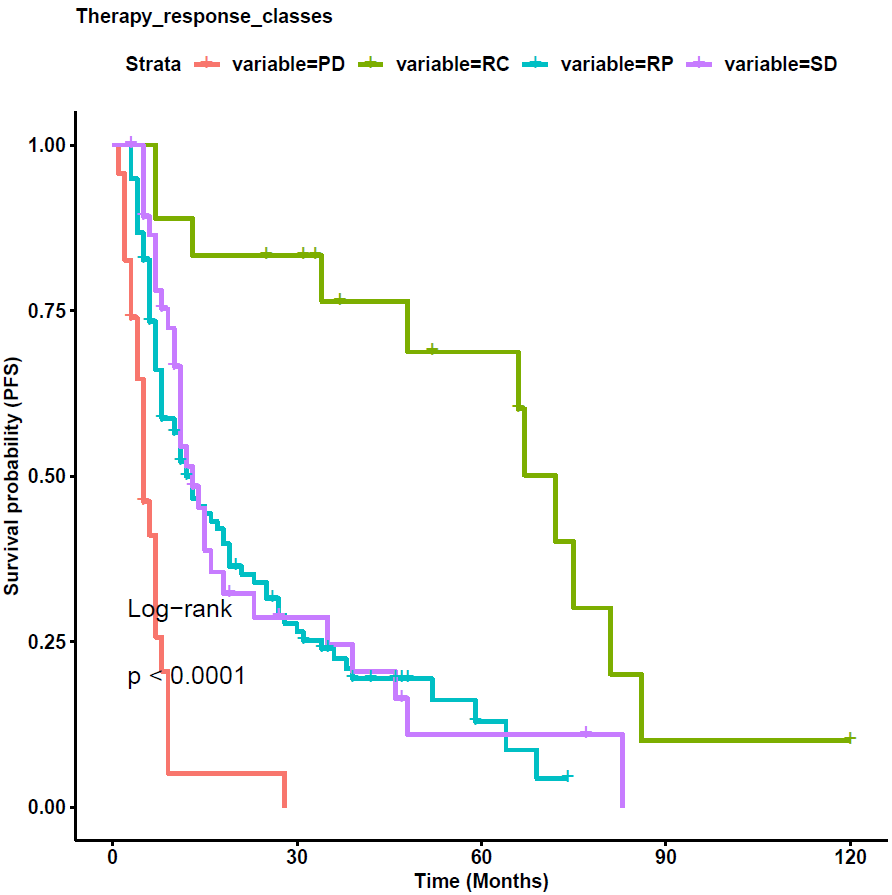
**

**Figure S2: Trend in Outcomes Based on Type of Response to BRAF-MEK Inhibitors in All Patients**

A total of 177 patients were classified into four groups: one group comprising non-responder patients (Progressive Disease, PD; 23 patients, red curve), one group including responder patients with Partial Response (RP; 98 patients, cyan curve), one group including responder patients with Complete Response (RC; 18 patients, green curve), and one group including responder patients with Stable Disease (SD; 38 patients, violet curve). The correlation between the variable value and patient survival was assessed in terms of Overall Survival (OS) [panel A] and Progression-Free Survival (PFS) [panel B]. Prognoses for each patient group were evaluated using Kaplan-Meier survival estimators, and survival outcomes were compared using log-rank tests. Log-rank p-values of ≤ 0.05 were considered statistically significant. Patients in the responder classes demonstrated a more favorable prognosis compared to those in the non-responder class, both regarding OS and PFS.

**Figures S3 and S4: Trends in Outcomes Based on DDI-H vs DDI-L in Responders and Non-Responders**

**Figure S3:** A total of 154 responder patients were classified into two groups: one group comprising patients with Drug Pin Light High (indicated by dark yellow, orange, or red; 27 patients, red curve) and another group comprising patients with Drug Pin Light Low (indicated by green or yellow; 127 patients, cyan curve). The correlation between the variable value and patient survival was assessed in terms of Overall Survival (OS) [panel A] and Progression-Free Survival (PFS) [panel B]. Prognoses for each patient group were evaluated using Kaplan-Meier survival estimators, and survival outcomes were compared using log-rank tests. Log-rank p-values of ≤ 0.05 were deemed statistically significant.

**Figure S4:** A total of 23 non-responder patients were classified into two groups: one group comprising patients with Drug Pin Light High (indicated by dark yellow, orange, or red; 10 patients, red curve) and another group comprising patients with Drug Pin Light Low (indicated by green or yellow; 13 patients, cyan curve). The correlation between the variable value and patient survival was assessed in terms of Overall Survival (OS) [panel A] and Progression-Free Survival (PFS) [panel B]. Prognoses for each patient group were evaluated using Kaplan-Meier survival estimators, and survival outcomes were compared using log-rank tests. Log-rank p-values of ≤ 0.05 were deemed statistically significant.

**Figure S5: Trend in Outcomes Based on DDI-H vs DDI-L in Low-Risk Patients**

A total of 78 low-risk patients were classified into two groups: one group comprising patients with Drug Pin Light High (indicated by dark yellow, orange, or red; 15 patients, red curve) and another group comprising patients with Drug Pin Light Low (indicated by green or yellow; 63 patients, cyan curve). The correlation between the variable value and patient survival was assessed in terms of Overall Survival (OS) [panel A] and Progression-Free Survival (PFS) [panel B]. Prognoses for each patient group were evaluated using Kaplan-Meier survival estimators, and survival outcomes were compared using log-rank tests.

Tables S1 e S2 (STAVANO NELLA TESI PER ME POSSONO ESSERE TOLTE… non sono fondamentali
