# Supplementary material for: Impact of Drug–Drug Interactions on Clinical Outcomes in Metastatic Melanoma Patients Treated With Combined BRAF/MEK Inhibitors: A Real‐World Study
Source: Pigment Cell Melanoma Res. 2025 Jun 1;38(4):e70026. doi: 10.1111/pcmr.70026 (PMC12127241; doi:10.1111/pcmr.70026)
Supplement: Supplementary file 2 — Appendix S2. [file PCMR-38-0-s001.docx]

**Table S1. Concomitant medications before starting treatment with anti-BRAFi/MEKi.**

| class of drug | patients |
| --- | --- |
| ACE inhibitors | 37 |
| diuretics | 20 |
| beta-blockers | 20 |
| sartans | 19 |
| calcium antagonist | 18 |
| antiplatelet | 17 |
| Proton pump inhibitors | 13 |
| statins | 12 |
| oral antidiabetics | 11 |
| corticosteroids | 10 |
| thyroid hormone | 9 |
| insulin | 9 |
| benzodiazePINe | 9 |
| alfa-blockers | 8 |
| antiepileptic | 7 |
| antidepressants | 4 |
| anti-coagulants | 3 |
| anti-arRhYtmic | 3 |
| anti-viral | 2 |
| low molecular weight heparin | 1 |

**Table S2. Drug-PIN light before and after the start of the concomitant BRAFi/ MEKi therapy.**

| Drug-PIN light | pre-anti-braf/mek | post-anti-braf/mek |
| --- | --- | --- |
| green | 157 | 112 |
| yellow | 10 | 28 |
| dark yellow | 8 | 22 |
| orange | 1 | 2 |
| red | 1 | 13 |

**
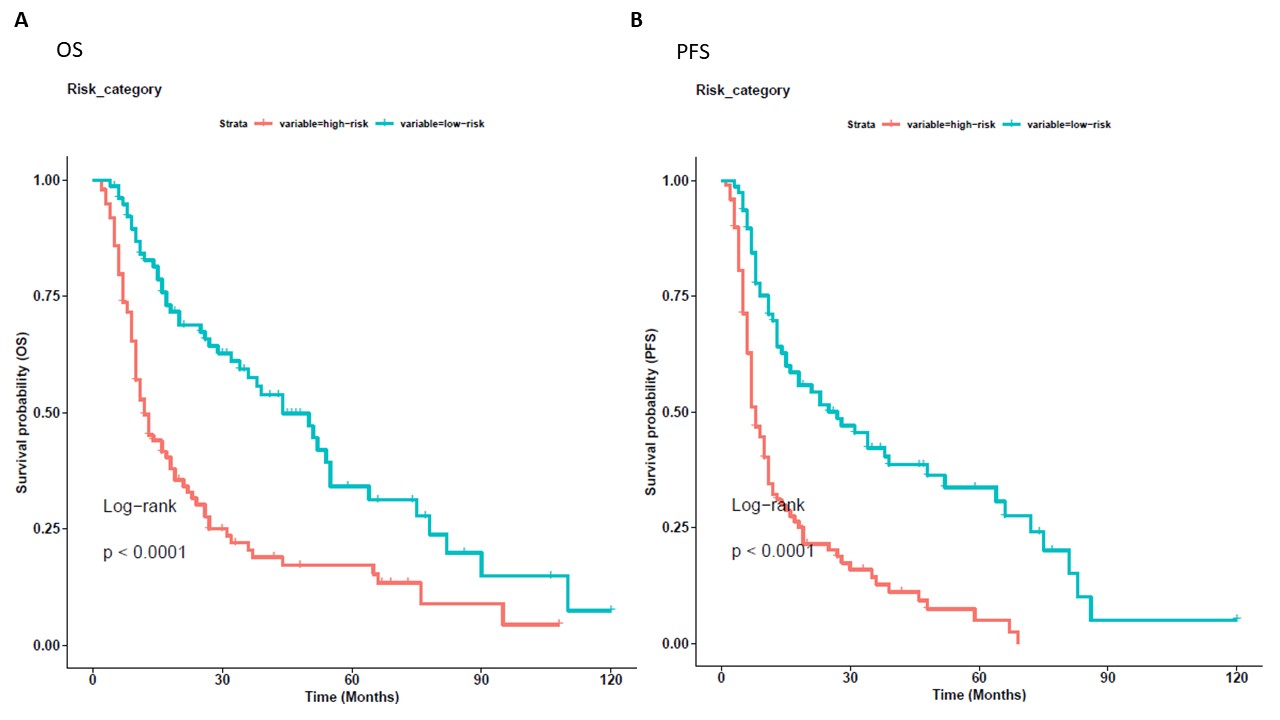
**

**Figure S1. Kaplan-Meier analysis.** 177 patients were classified into two groups: one class including patients with high risk (99 patients, red curve), and the other one including patients with low risk (78 patients, cyan curve) . The correlation between variable value and patient survival was examined as OS [panel A] and PFS [panel B]. The prognosis of each group of patients was examined by Kaplan-Meier survival estimators, and the survival outcomes of the two groups were compared by log-rank tests. Log rank p-values less than or equal to 0.05 were considered as statistically significant. Patients belonging to low risk class show a good prognosis with respect to the ones belonging to high risk class both in terms of OS and PFS.

**
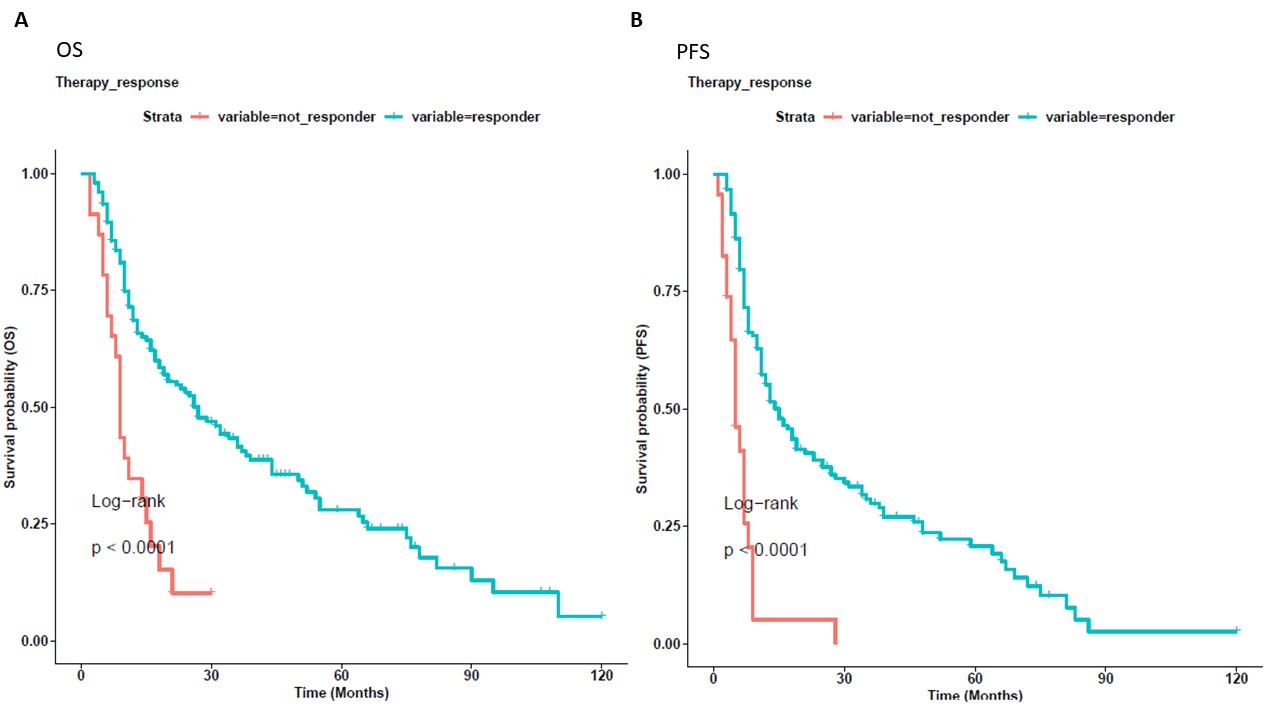
**

**Figure S2. Kaplan-Meier analysis.** 177 patients were classified into two groups: one class including non-responder patients (23 patients, red curve), and the other one including responder patients (154 patients, cyan curve). The correlation between variable value and patient survival was examined as OS [panel A] and PFS [panel B]. The prognosis of each group of patients was examined by Kaplan-Meier survival estimators, and the survival outcomes of the two groups were compared by log-rank tests. Log rank p-values less than or equal to 0.05 were considered as statistically significant. Responder patients (i.e. reporting SD, RP or CR as best response) had significantly better OS (19 months (3-120), HR 0.31, CI 95% (0.19-0.51), p-value=4,94E-06) and PFS (9 months (2-30), HR = 0.22 , CI 95% (0.13-0.36), p-value = 2,01E-09) than non-responder patients (i.e. reporting PD as best response).


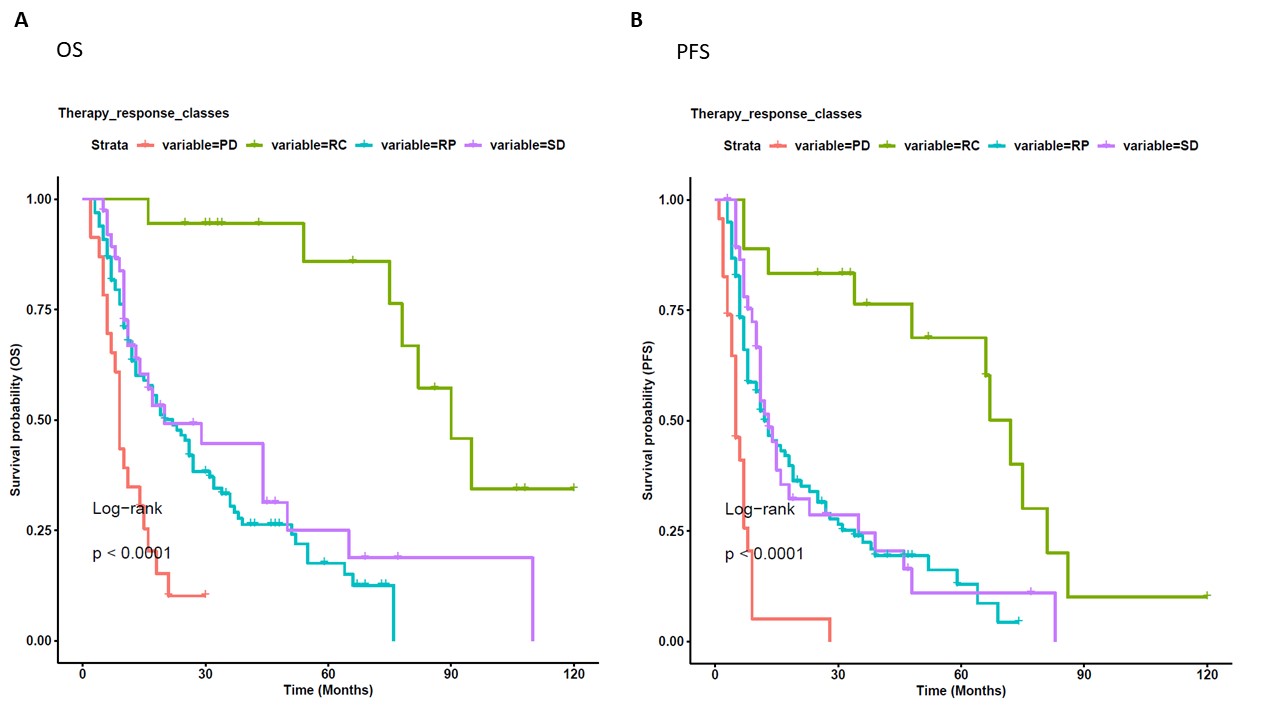


**Figure S3**. 177 patients were classified into 4 groups: one class including non-responder patients (PD, 23 patients, red curve), one including patients achieving RP (98 patients, cyan curve), one including responder patients achieving RC (18 patients, green curve), one including patients achieving SD (38 patients, violet curve). The correlation between variable value and patient survival was examined as OS [panel A] and PFS [panel B]. The prognosis of each group of patients was examined by Kaplan-Meier survival estimators, and the survival outcomes of the two groups were compared by log-rank tests. Log rank p-values less than or equal to 0.05 were considered as statistically significant. Responder patients had a better OS and PFS (p<0.0001), compared to non-responder patients, with the best outcome reported by patients achieving complete response (median OS 70.5 (16-120), HR = 0.153, CI 95% (0.067-0.35), p-value=8,14E-06). Median OS was 18 (2-30), 13.5 (13-120) and 9 months (2-30) in patients achieving PR (HR = 1.42, CI 95% (0.97-2.1), p-value 0.068), SD (HR = 0.92, CI 95% (0.59-1.4), p-value 7,31E-01) and PD (HR = 3.23, CI 95% (2-5.3), p-value 4,94E-06), respectively. Similarly, the best PFS was reported in patients achieving RC (50 months (7-120), HR = 0.253, CI 05% (0.13-0.49), p-value= 5,60E-05), while the median PFS was 11 (1-28), 11 (7-120) and 5 months (1-28) in patients with PR (HR = 1.18, CI 95% (0.83-1.7), p-value= 0.358), SD (HR= 0.95, CI 95% (0.63-1.4), p-value=8,24E-01) and PD (HR = 4.53, CI 95% (2.8-7.4), p-value= 2,01E-09), respectively.
